# Improved Self-Esteem in Artists After Participating in the “Building Confidence and Self-Esteem Toolbox Workshop”

**DOI:** 10.3389/fpsyg.2018.01169

**Published:** 2018-07-05

**Authors:** Anita R. Shack, Soumia Meiyappan, Loren D. Grossman

**Affiliations:** ^1^Al and Malka Green Artists' Health Centre, Toronto Western Hospital, University Health Network, Toronto, ON, Canada; ^2^Department of Family and Community Medicine, Toronto Western Hospital, University Health Network, Toronto, ON, Canada; ^3^Faculty of Medicine, University of Toronto, Toronto, ON, Canada

**Keywords:** self-esteem, artists, workshop, performance, health, transformation

## Abstract

Performing and creative artists have unique occupational and lifestyle stresses and challenges that can negatively affect self-esteem. Low self-esteem not only has serious implications for their psychological and physical health, it can also affect their performance, and creativity. There is a need to establish effective interventions to deal with this issue. To the best of our knowledge, there are no reported studies specific to workshops or interventions on enhancing self-esteem for artists. The Al and Malka Green Artists' Health Centre at the Toronto Western Hospital, University Health Network, Toronto, Ontario, Canada, is a unique multidisciplinary, and integrative clinic serving the special needs of the artist population. We developed a workshop entitled “Building Confidence and Self Esteem Toolbox Workshop” to address this need. We then designed a single-blind, randomized, prospective, pilot study to evaluate the effectiveness of the workshop on enhancing self-esteem in artists, and to evaluate the long-term effectiveness of using the recommended tools in maintaining a healthy self-esteem, as well as maintaining physical and emotional health. Both quantitative and qualitative data were collected. A validated “Self-Esteem Checkup” questionnaire was administered pre- and immediately post workshop, as well as at 2, 6, and 12 months post workshop. Open-ended questions were posed to study participants via email at 2 and 12 months following the workshop, and at 6 months in in-person interviews. Thirty-five professional artists consented to participate in the study, with 26 completing all study visits. Mean scores for all time points, and the individual questionnaire statement mean scores for the five timepoints increased significantly post-workshop and remained statistically significantly improved by the 3rd follow-up 12 months later (*p* < 0.001). The mean self-esteem rating score increased significantly post-workshop and remained statistically significantly improved by the 3rd follow-up 12 months later (*p* < 0.01). Qualitative data showed positive feedback on the utilization of the tools learned in the workshop that helped maintain this improvement over a 1-year period. This workshop may be an effective means of addressing the issue of self-esteem in artists. Further controlled studies of larger sample size and longer duration are needed to confirm these findings.

## Introduction

Healthy self-esteem, defined as a realistic appreciation of oneself (Schiraldi, 2001), is an important element of psychological and physical health (Hudd et al., 2000; Kenny, 2011; Koban et al., 2017; Buckley, 2018). It serves as a basis and motivation for behavior that encodes positive attitudes, perceptions and self-talk, and provides ways to buffer stress and stabilize oneself in times of intense stress or negative feedback (Pyszczynski et al., 2004; Creswell et al., 2013). Self-confidence is a narrower construct that can be defined as simply believing in oneself (Blascovich and Tomaka, 1991; Benabou and Tirole, 2002).

The association between low self-esteem and stress and increased anxiety, and between high self-esteem and wellbeing are supported in the literature (Abouserie, 1994; Hudd et al., 2000; Sakellari et al., 2018). Negative self-esteem has been associated with serious health issues including eating disorders, social anxiety disorder, and suicide (Kelly et al., 2014; Ianucu et al., 2015; Eades et al., 2018). Elevated levels of stress and low self-esteem can impact one's ability to function, perform, and create (Steptoe, 2001; Brady, 2011). This has significant implications for artists across all disciplines.

Artists have unique occupational and lifestyle stresses and challenges that can negatively impact self-esteem. In addition to dealing with the demands of daily life (interpersonal relationships, finances, time management), they are under pressure to train, practice, perform, produce, create and to cope (van Fenema and van Geel, 2014; Vervainioti and Alexopoulos, 2015). Low self-esteem is a factor in performance anxiety (Langendorfer et al., 2006). This extreme stress experienced by musicians, dancer, and actors can be debilitating and career shortening if not career ending in its effect (Miller and Chesky, 2004; Zwaan et al., 2010). Many artists need to promote and produce their own work. Many are underemployed and/or undercompensated for their artistic work. Low financial status can negatively impact self-esteem (Fabiani et al., 2016; Gardner et al., 2016). The demands on the bodies, minds, and emotions of artists poses health risks and can lead to injuries (Shrier and Hallé, 2011; Rickert et al., 2013). Coping with compromised health and injuries leads to further stresses (O'Dea and Abraham, 2000). Lack of social support from peers, families, friends, educators, and health care providers, is another stress factor contributing to low self-esteem (DeLongis et al., 1988).

Artists' identities are tightly bound to their artistic discipline and pursuits (Parncutt and McPherson, 2002). They live in a world where comparison, competition, and perfectionism are widespread (Mor et al., 1995; Langendorfer et al., 2006; Nordin and McGill, 2009). Literature supports the belief that professional performing and creative artists share a tendency to perfectionism that is associated with low self-esteem and low self-concept, and higher levels of anxiety (Marchant-Haycox and Wilson, 1992; Kenny et al., 2004; Hamilton and Robson, 2006; Bodner and Bensimon, 2008; De Haas and Winterkorn-Pierrot, 2009; Nordin-Bates et al., 2011; Schellenberg, 2011; Simmonds and Southcott, 2012). Studies of music and dance students also support this view (Nordin and McGill, 2009; van Fenema and van Geel, 2014). Those who set excessively high standards for themselves tend to be over critical in self-evaluation, feeling they are never good enough, self-doubt, and anxiety over mistakes (van Fenema and van Geel, 2014). Often being subjected to the extrinsic factors of correction, criticism, judgement, and rejection from family, educators, audiences, critics, agents, curators, producers, directors, choreographers, and peers is part of the artists' reality. This can erode self-esteem and contribute to creating a strong voice to artists' negative inner dialogue. Negative self-talk can seriously affect performance (Parncutt and McPherson, 2002).

Clearly there is a pressing need for healthy coping strategies, empowering self-evaluation tools, support, education, and interventions aimed at addressing the issue of self-esteem in the artist population. The benefits of improved self-esteem for artists can result in a better ability to manage stress, improved physical, and mental health, and enhanced ability to create and perform (Nordin and McGill, 2009).

The Al and Malka Green Artists' Health Centre at the Toronto Western Hospital, University Health Network, Toronto, Ontario, Canada, is a unique multidisciplinary, and integrative clinic. It includes natural and traditional medicine practitioners in a collaborative, holistic, patient centered model serving the special needs of the artist population. This includes performing and creative professional artists from all disciplines (music, theater, dance, film, writing, circus, photography, visual artists, etc.). Over the course of more than 15 years working with these artists, we have observed that many presenting to the clinic for either stress related emotional, psychological or musculoskeletal complaints, had underlying issues of low self-esteem. Educational group workshops designed to address the requests and perceived needs of the artist population are part of the mandate of this clinic. In 2009 there was a request for a workshop to help address self-esteem.

We developed a workshop entitled “Building Confidence and Self Esteem Toolbox Workshop” to address this need. The design of the workshop was intentionally created to take the participants through a transformative experiential journey to facilitate meaningful change. Theoretical knowledge and concepts were taught and discussed to further enhance understanding, help shift view points and belief systems, and support the steps in the experiential process (Branden, 1994; Strikes and Orsi, 1999; Schiraldi, 2001; Kukla, 2007).

The issue of self-esteem is complex and impacts a person in all aspects; physical, emotional, mental, and spiritual. Therefore, a variety of exercises and experiences were included to affect the participants on these levels. Some of these included guided imagery, meditation, writing, partner work, and ceremonial ritual. As part of the journey, participants challenge their current beliefs, assumptions, and perceptions of negative self-esteem, explore where these come from, identify, and learn to manage personal obstacles to attaining a positive self-concept, discover tools to free themselves from being attached to the judgements of others, and to constructively self-evaluate, deal with negative thought patterns, and embrace positive self-esteem as a motivation for self-care.

The workshop begins with an introspective exercise where participants acknowledge and feel the current perception of their self-esteem. As they engage with the tools and teachings in the process of the journey, they increase awareness and clear negativity, which allows them to become receptive and ready to make empowered changes. They then encode and integrate the changes and shift from negative to positive self-esteem with a ceremonial ritual. The last step of the process revisits the first exercise so that participants can recognize the change that has transpired during the course of the workshop. As well, the workshop provides the participants with a set of tools they can continue to use after its completion, to maintain, and enhance their new level of self-esteem.

This workshop was offered at least 12 times during a 4 year period, each time modified according to participant feedback, until a consistent design and content were established. Based on positive feedback, we decided to research the effectiveness of the workshop. Beyond a positive experience of attending the workshop, we wanted to investigate whether attendees experienced lasting change in their perception of self-esteem, and what impact this change may have had on their creative processes and in their lives.

We designed a single-blind, randomized, prospective, pilot study to evaluate the effectiveness of the “Building Confidence and Self-Esteem Workshop” on enhancing self-esteem in artists, and to evaluate the long-term effectiveness of using the recommended tools in maintaining a healthy self-esteem, as well as maintaining physical and emotional health.

## Materials and methods

### Recruitment

The Artists' Health Alliance is an organization that provides health and wellness support for artists of all disciplines in Canada. It advertised two 6-hour Building Confidence and Self-Esteem Toolbox Workshops via email to their email list serve, consisting of individual performing, and creative artists and arts organizations and unions. Workshop attendees needed to meet the criteria of professional artists, as outlined in the Canadian Artist Code (www.csarn.ca). The first 20 attendees who randomly registered for each workshop were accepted. Study subjects were randomly recruited to participate in the research study in a single-blinded fashion. Two envelopes were distributed to each workshop attendee, each containing a copy of the study consent form and a participant contact sheet. Each pair of envelopes was labeled with a pre-assigned study ID number. If a workshop attendee was interested in participating in the research study, she/he was instructed to sign both copies of the consent form and fill in both copies of the participant contact sheet, place each copy in its respective envelope and return one sealed envelope to the study coordinator. The workshop facilitators/investigators were blinded to this process so that they were unaware of which workshop attendees were participating in the study.

### Data collection

Both quantitative and qualitative data were collected for this study. As such, a Concurrent Triangulation Strategy Mixed Methods Design was used (Creswell, 2003), in which we collected quantitative and qualitative data concurrently at each of the three follow-up time-points of this study.

### Quantitative data

A validated “Self-Esteem Checkup” questionnaire was administered pre- and immediately post workshop, as well as at 2, 6, and 12 months post workshop (Schiraldi, 2001). The questionnaire aims to measure how the respondent feels at the present moment. Respondents were to rate themselves on a scale from 0 to 10 for each of the 12 statements in the questionnaire. The questionnaire also contained two closed-ended Likert scale items assessing an overall self-esteem rating score (0–100) and how restricted they felt in their daily activities because of difficulties with self-esteem (1–5) (**Appendix 1**).

### Qualitative data

Two open-ended questions were posed to study participants via email at 2 and 12 months following the workshop (Table 1). The qualitative data was gathered by a study coordinator who was not one of the workshop facilitators. Open-ended questions were posed with answers transcribed by the study coordinator without prompting.

**Table 1:** Open-ended questions posed at 2 and 12 months.

1. Do you feel that your work as an artist has been influenced by your participation in the workshop?
a) If yes, how?
b) If no, why not?
2. In what ways do you think the workshop has impacted your physical and emotional health (both positively and negatively)?

At 6 months participants were invited for in-person interviews (Table 2).

**Table 2:** Interview Questions at 6 months.

1) What self-esteem workshop tools have you found to be the most helpful?
2) Which tools are you using on a regular basis?
a. Why, or why not?
3) What do you think would assist you to better utilize the tools presented at the workshop?
4) Do you feel that your work as an artist has been influenced by your participation in the workshop?
a. If yes, How?
b. If no, why not?
5) In what ways do you think the workshop has impacted your physical and emotional health? (Both positively and negatively).
6) Any other comments?

### Data analysis

A mean ± SEM score of the 12 responses in the questionnaire was calculated for the participants for each questionnaire they completed. As well, the mean response for each statement was followed over the five time points: pre-workshop, immediately post-workshop, 2, 6, and 12 months post-workshop. Changes in mean scores were analyzed by a repeated measures ANOVA using SPSS 21.0. Descriptive statistics assessed normality of the data.

Responses to the open-ended questions were listed in tabular format, divided into “Yes, how?” and “No, why not?” for the first question, and categorized as positive impacts and negative impacts that the workshop has had on one's physical and emotional health for the second question, and then subsequently collapsed into more defined categories. These categories were combined into general themes with illustrative quotes.

Individual interview question responses were transcribed and coded after becoming familiar with the responses, rather than using predetermined categories. These codes and categories identified within the responses were sorted and collapsed into broader themes (Krefting, 1991).

### Ethics

This study was approved by the University Health Network Research Ethics Board and written informed consent was obtained from each subject prior to any study procedures being carried out. The study was conducted in accordance with ICH guidelines.

## Results

Two workshops were run with study participants included. A total of 40 subjects registered for the two workshops. Nineteen from the first and 16 from the second, for a total of 35 subjects attended and consented to participate in the study. Sixteen subjects from the first, and 10 subjects from the second, for a total of 26 subjects completed all study visits. Nine subjects did not return email responses to the follow-up questionnaires.

### Quantitative data

**Figure 1:**
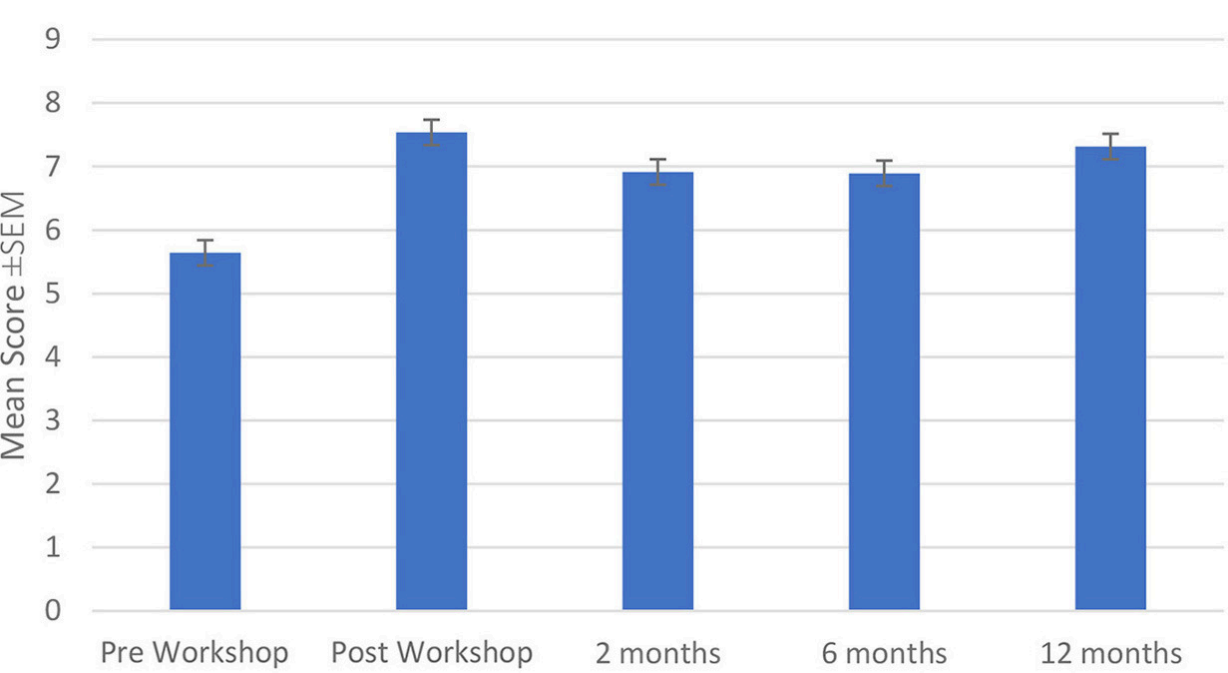
Mean questionnaire scores.

The mean ± SEM scores for the 12 questionnaire statements are shown in Figure 1. The pre-workshop mean score was 5.64 ± 0.31. All scores increased significantly immediately post-workshop and remained statistically significantly improved by the by the 3rd follow-up 12 months later. Post-workshop, the mean score was 7.54 ± 0.19, at 2 months 6.92 ± 0.19, at 6 months 6.89 ± 0.23, and at 12 months post-workshop the mean score was 7.32 ± 0.22 (*p* < 0.001 for all post-workshop times compared with pre-workshop).

**Figure 2:**
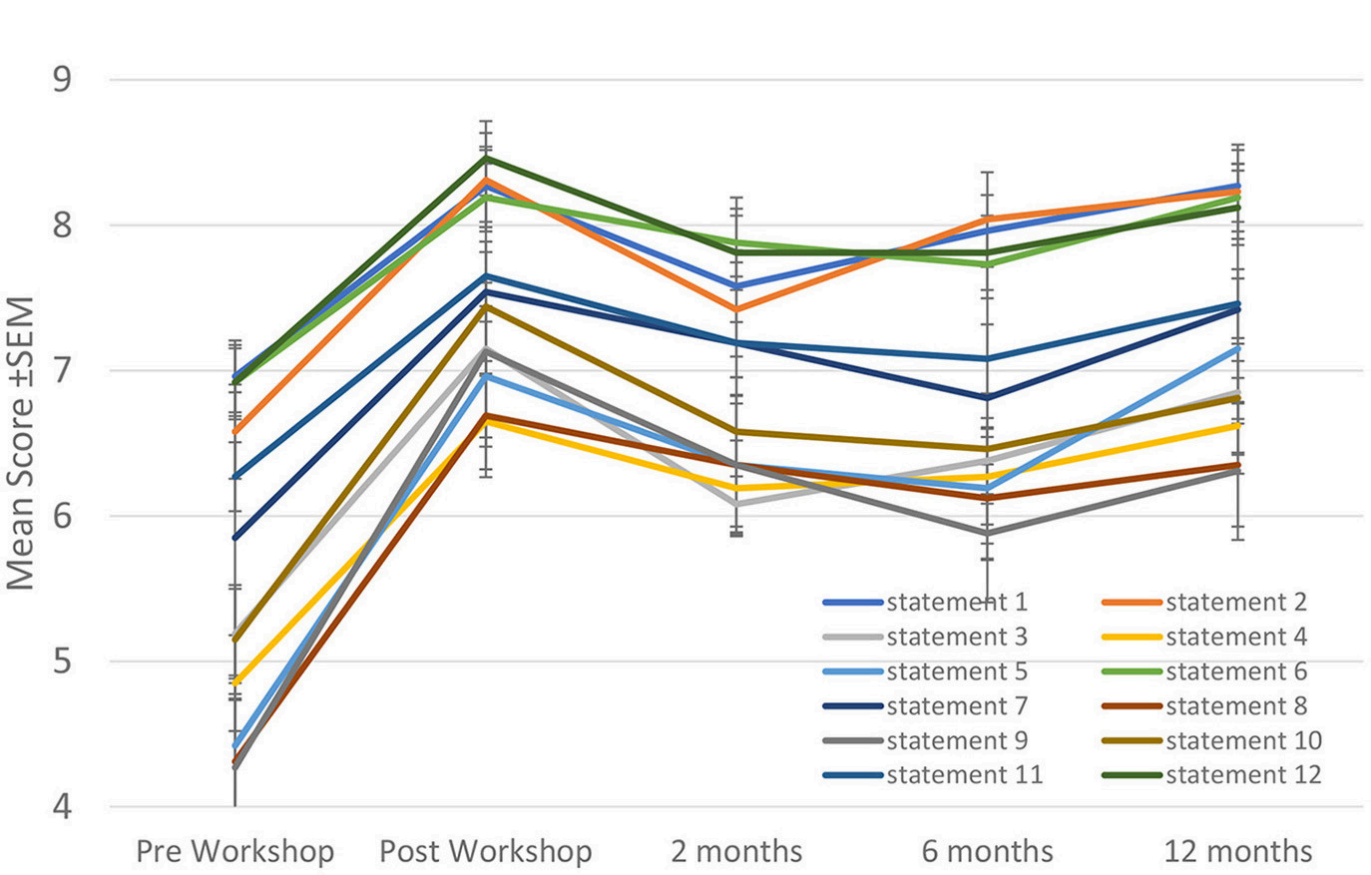
Self-esteem questionnaire individual statement scores.

The individual questionnaire statement mean ± SEM scores for the five timepoints are shown in Figure 2. All scores increased significantly immediately post-workshop and remained statistically significantly improved by the by the 3rd follow-up 12 months later (*p* < 0.001 for all time points compared to pre-workshop). Post-workshop scores were not significantly different from each other. Cronbach's alpha pre-workshop was 0.933, immediately post-workshop was 0.949, at 2 months 0.965, at 6 months 0.579, and at 12 months 0.545.

**Figure 3:**
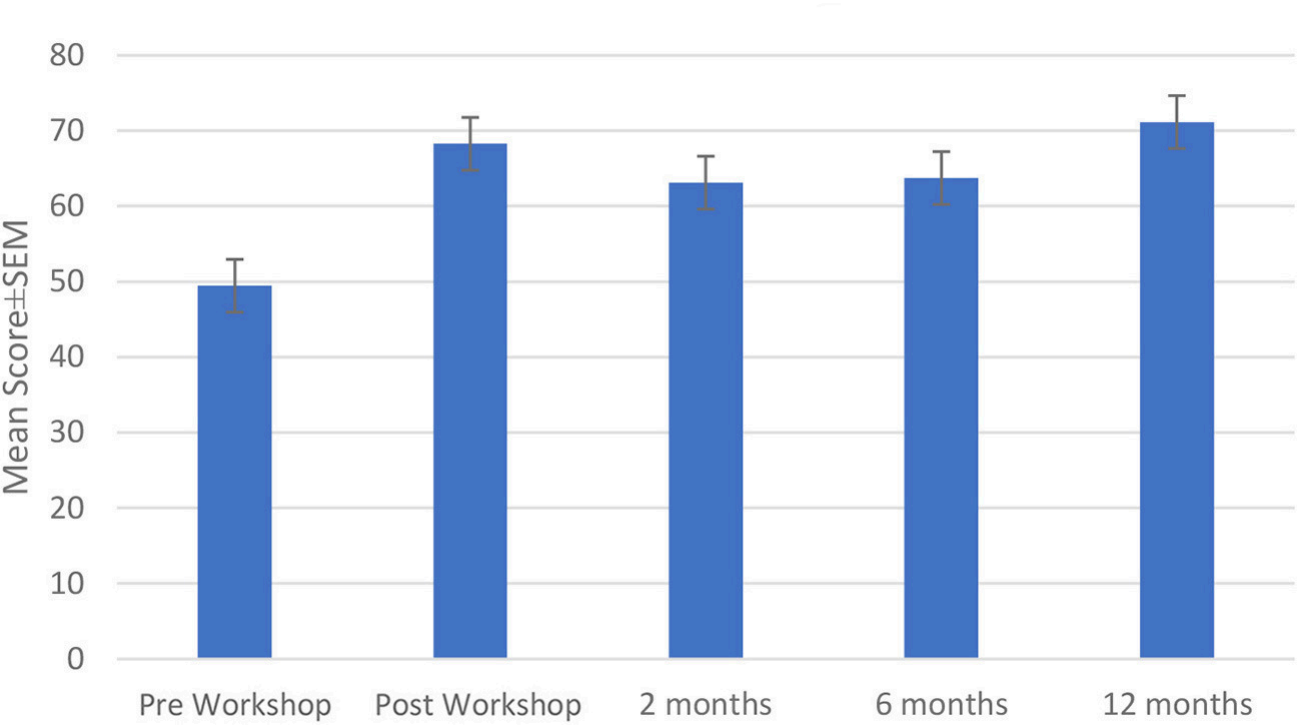
Mean self-esteem rating scores.

The mean Self-Esteem Ratings (0–100 scale) are shown in Figure 3. The mean pre-workshop score for the participants was 49.46 ± 3.37. All scores increased significantly immediately post-workshop and remained statistically significantly improved by the 3rd follow-up 12 months later. Post-workshop, the mean score was 68.27 ± 3.51, at 2 months 63.12 ± 3.40, at 6 months 63.73 ± 4.39, and at 12 months post-workshop the mean score was 71.15 ± 3.61, (*p* < 0.01 for all post-workshop times compared with pre-workshop). Post-workshop scores were not significantly different from each other.

For the question on restriction of daily activities, there was no significant difference in responses post-workshop compared with pre-workshop.

### Qualitative data

Responses to the open ended questions were analyzed and categorized into five primary themes: group setting effective mode of presentation, positive impact on performance and creativity, transformational learning experience, integrative culminating experience, and request for refresher workshops.

#### Theme 1: group setting effective mode of presentation

The group setting of the workshop provided reassurance that peers faced similar struggles as illustrated in the following quotes: “*The whole sitting around in a group, and hearing other people going through the same things that you're going through, is very, very therapeutic. Like, to me, that is a tool in itself*.” and “*I was quite pleased to be considered one of their peers. And realizing just how common this is. People that I would consider intimidating can struggle with the exact same problems*.”

#### Theme 2: positive impact on performance

Participants remarked on the positive impact the workshop had on their performance and how it motivated them to take risks that they might not have taken before the workshop: “*I already was booked up that day to shoot a video and I normally would not have put in my audition, but I went for it. I might not have done that. I just have to push myself to do it. My resume has bumped up a lot*.” Feelings of optimism and promise following the workshop were also shared: “*I know I was feeling more hopeful. I think it was the idea of possibilities*.” “*I remember [workshop facilitator] had said, you know, even if I didn't get the part, I still went. And I remember I was using that for a while. And actually, I think that's maybe permeated more than I thought into my life…where I just kinda go, “you know what? I did something.” but it's like, you gotta do the little things, and so as long as you do something, it's better than nothing.”* “*Well, it's nice to voice, yeah, where I am and, and what I want to do. It's frustrating to come back to wanting to do the same thing when I've come back to wanting to do the same thing before. It struck me that I had progressed more than I would have thought because I'd sort of, in my mind, felt I hadn't done anything, but actually, we did do a mockup of two, two books.”*

#### Theme 3: transformational learning experience

Participants engaged in a partnered exercise where they had the opportunity to retell a story and allow their past perceptions of an experience to change. This was described as a transformational learning experience. Noted one participant: “*The idea that you could rewrite your own story to have a different ending. Just sort of seeing it in a different perspective.”*

Another participant reflected on her newfound ability to disassociate the results of her work from her self-perception: “*Remembering that the criticisms that other people have really have to do with them. It's that thinking well, okay, I struggle at this but, I still am a good person.”*

#### Theme 4: integrative culminating experience

Within the workshop, participants track and record what they perceive as their negative and positive attitudes, traits, and patterns. At the end of the workshop, in a ritualized experiential exercise, they throw away the negative thoughts they have recorded, and choose to pick up and keep the positive ones to review. As one participant replied: “*you're writing out your negative pieces, and then, you throw it away and bring in your strengths, the pieces you wanna go forward. Seeing each piece and purging in some ways*.” Another said “*Sometimes you don't know how much that will affect you until you do it. Cause it sounds, you know, ‘okay, we're gonna put them in a…in a little pot' but it actually, it actually is very helpful in a weird way*.”

#### Theme 5: request for refresher workshops

Participants expressed an interest in some type of follow-up sessions, thinking this would help support them in continuing the improvements they had learned. Three participants commented: “*But in a perfect world, it could be a weekly thing where you go and it's like a maintenance thing, kind of keep in check…all your tools, all your, the things that you've learned.”* “*It would have been great to have a check-in to prolong that effect; The workshop was not long enough…”* “*I don't know if this would work, especially since people have such busy schedules, but something that was over a longer period of time ? Or having…not necessarily every week but…like a refresher? I don't know. Like, maybe in a couple of months, or something…even though it was a very intensive."*

The experiential tools that were reported to be most useful in the workshops included:

Change That Story-a partner exercise where one person listens and the other repeats a personal story with the same details as many times as it takes until there is a shift in perception and experience from negative to positive.

Breathing and Guided Mediation Journey-allowing physical relaxation and suggestion to engage the participant where patterns are addressed at an unconscious and spiritual level.

Light and Dark Arrow Ritual-a group ceremony where there is integration of the workshop's experiences and learning. In an empowering act, each individual literally shoots their dark arrow and picks up a light arrow to encode and embody their changes.

Stop That Thought-a cognitive behavioral therapy exercise to enhance awareness of automatic thoughts and to allow for a new thought pattern.

Self-Care Strategies-a group discussion where behaviors are examined in the light of self-care and a useable menu of tools is created.

## Discussion

The results of our study suggest that the “Building Confidence and Self-Esteem Toolbox Workshop” was effective in increasing the self-esteem scores of the artist participants. They utilized tools learned in the workshop that helped them maintain this improvement over a 1-year period. Self-esteem scores improved immediately post-workshop, and remained elevated compared to pre-workshop, at 2, 6, and 12 months post-workshop. This workshop may be an effective means of addressing the issue of self-esteem in artists.

The themes that emerged from the qualitative data analysis provide further insights into some of the more effective components of the workshop.

### Group setting

Although some artistic disciplines are more socially interactive, many artists work in isolation, such as writers and visual artists. Even those who are in more social settings such as dancers, actors, and musicians can also feel isolated due to an environment of competition and comparison. Coming together with others to share in an equal way, with the same intention, in an atmosphere of trust was deemed to be therapeutic in of itself. Many respondents suggested continuing with an ongoing support group. Social isolation has been shown to be associated with low self-esteem (Leary, 1990; Leary et al., 1995).

### Positive impact on performance and creativity

The emergence of this theme suggests that participants experienced an internal shift as a result of the workshop, that leads to changes in behavior, improved self-esteem, and enhanced performance and creativity. Anxiety reducing techniques have been shown to increase performance (Holland et al., 2017).

### Transformational learning experience

This theme refers to the empowerment of the “Change That Story” exercise in the workshop. This provided an opportunity to relate a personal story to a neutral listener and have the perception of self-esteem within that story change from negative to positive. This exercise requires commitment and a degree of trust due to the intimacy involved. Participants were surprised that such a profound change could occur in such a short period of time.

### Integrative culminating experience

Participants appreciated this last element of the workshop design as a way of making the new insights and experiences physically realized with an empowering act and conscious intention to let go of the negative and embrace the positive. Creative process was engaged in making dark and light arrows imbued with meaning. Involving all the senses in this ceremonial ritual impacted the body mind and spirit of the participants in profound ways (Laird, 1984).

### Request for refresher workshops

Due to the nature of this research pilot project, granting this request would have been inappropriate until the research project was completed. Despite this theme, results supported a lasting effect out to 1 year.

To the best of our knowledge, there are no reported studies specific to workshops or interventions on enhancing self-esteem for artists. There is however, support for the need for such interventions (Mann et al., 2004; Vervainioti and Alexopoulos, 2015). Indeed, in a study of dancers, the authors conclude: “*dancers would benefit from programs that enhance self-esteem and reduce the negative effects of internalized shame and self-oriented and socially prescribed perfectionism*” (Eusanio et al., 2014).

The “Building Confidence and Self-Esteem Toolbox Workshop” was designed as an intervention to guide the artist participants in a transformative process, wherein their concepts of self could be explored and changed. The results of our study demonstrate that a shift in perception from negative to positive self-esteem can be achieved and maintained.

Many of the components, tools, and methods employed in the workshop are supported by previous studies demonstrating their ability to positively affect self-esteem. These include cognitive behavioral therapy (Thurston et al., 2017), meditation (Dahl et al., 2015; Fabiani et al., 2016; Hankey and Shetkar, 2016; Xiao et al., 2017), creative visualization (Fiske, 2017), introspective reflection and writing (Lauer and Goldfield, 1970; Chandler, 1990), self-care mindfulness (Adimando, 2017), ceremonial ritual (Wozniak and Allen, 2012), and self-compassion and mindfulness (Galla, 2016).

There is also evidence to support the overall design and mind/body approach of the workshop (Tarantino et al., 2013; Pai, 2016; Rodkjaer et al., 2017). This intermodal intervention included synergistic use of therapeutic devices, in a safe and empathetic atmosphere which was conducive to self-learning and was reported by some participants to be a healing experience (Martin et al., 2011; Jansen and Lang, 2012).

## Limitations of the study

This was a pilot study to evaluate the effectiveness of the workshop, with a small sample size. With no previous publications of a similar workshop available, it was difficult to estimate the potential benefit and perform a power calculation to determine the appropriate sample size necessary. Thus, a larger study will be necessary to confirm these findings. As well, this was a single blind uncontrolled study where the participants were aware they were enrolled in a study, and there was no control group. Study effect could have played a role in the results. Subject participation was solicited from an email mailing list rather than totally unsolicited participation, so that selection bias may have also played a role in the results. One year follow-up may be insufficient to determine a lasting effect on self-esteem, so longer-term follow-up may be necessary to confirm the findings.

### Future directions

Although the quantitative results were highly statistically significant, a longer-term controlled study with a larger sample size should be conducted to confirm and expand on the results. With a larger sample size, a breakdown of the different artistic disciplines might shed light on differences among the different types of artists studied, so that future interventions could be more targeted if necessary. A double blind controlled study design would further reduce biases. Subsequent studies can include more detailed questioning targeting improved overall health, performance, and creativity in addition to self-esteem scores. Larger scale studies should include a validated questionnaire such as the Rosenberg Self-Esteem Scale. Given the importance of performance anxiety in artists, and its association with self-esteem, a more detailed assessment of the workshop's impact on performance anxiety would be of benefit. Future studies could include a controlled study in a larger population of artists such as a professional or university music or dance program, where some attend the workshop in their first year and others do not. All are then followed though their program for its long-term effect on their self-esteem, performance anxiety, and aspects of their emotional and physical health, and how this impacts their motivational climate (Miulli and Nordin-Bates, 2011). Once validated, many other possible applications for the workshop exist, such as inclusion of the workshop in curricula to mitigate negative consequences of low self-esteem in future artists.

## Author contributions

AS conceptualized and developed the design and content of the workshop, facilitated the workshops, conceptualized the study, and developed the study protocol, wrote, reviewed, and edited the manuscript. SM was study coordinator, obtained informed consent, conducted the qualitative interviews, performed the initial data analysis, and statistical calculations, and reviewed and edited the manuscript. LG performed further detailed data analyses of the quantitative data, further statistical analyses and graphing of the data, and wrote, reviewed, and edited the manuscript.

### Conflict of interest statement

The authors declare that the research was conducted in the absence of any commercial or financial relationships that could be construed as a potential conflict of interest.

## Appendix 1

### Self esteem checkup questionnaire

First, rate from 0 to 10 how much you believe each of the following statements. 0 means you completely disbelieve it. 10 means you think it is completely true.

**Table:**

1. I am a worthwhile person.	_______
2. I am as valuable a person as anyone else.	_______
3. I have the qualities I need to live well.	_______
4. When I look into my eyes in the mirror I have a pleasant feeling.	_______
5. I don't feel like an overall failure.	_______
6. I can laugh at myself.	_______
7. I am happy to be me.	_______
8. I am generally satisfied with the way I am developing as a person.	_______
11. I respect myself.	_______
12. I'd rather be me than someone else.	_______
Total Score	_______

Next, rate your self-esteem on the following scales (Gauthier et al., 1983):

How often do you feel restricted in your daily activities because of difficulties with self-esteem?

Reprinted with permission from Schiraldi. (2001). *The Self-Esteem Workbook*. Oakland, CA: New Harbinger. ©2001 Glenn R. Schiraldi, Ph.D. Not to be reproduced without written permission
